# Values of spleen-preserving distal pancreatectomy in well-differentiated non-functioning pancreatic neuroendocrine tumors: a comparative study

**DOI:** 10.1093/gastro/goac056

**Published:** 2022-10-13

**Authors:** Xi-Tai Huang, Jin-Zhao Xie, Jian-Peng Cai, Peng Fang, Chen-Song Huang, Wei Chen, Li-Jian Liang, Xiao-Yu Yin

**Affiliations:** Department of Pancreato-Biliary Surgery, The First Affiliated Hospital, Sun Yat-sen University, Guangzhou, Guangdong, P. R. China; Department of Pancreato-Biliary Surgery, The First Affiliated Hospital, Sun Yat-sen University, Guangzhou, Guangdong, P. R. China; Department of Pancreato-Biliary Surgery, The First Affiliated Hospital, Sun Yat-sen University, Guangzhou, Guangdong, P. R. China; Department of Pancreato-Biliary Surgery, The First Affiliated Hospital, Sun Yat-sen University, Guangzhou, Guangdong, P. R. China; Department of Pancreato-Biliary Surgery, The First Affiliated Hospital, Sun Yat-sen University, Guangzhou, Guangdong, P. R. China; Department of Pancreato-Biliary Surgery, The First Affiliated Hospital, Sun Yat-sen University, Guangzhou, Guangdong, P. R. China; Department of Pancreato-Biliary Surgery, The First Affiliated Hospital, Sun Yat-sen University, Guangzhou, Guangdong, P. R. China; Department of Pancreato-Biliary Surgery, The First Affiliated Hospital, Sun Yat-sen University, Guangzhou, Guangdong, P. R. China

**Keywords:** pancreatic neuroendocrine tumor, distal pancreatectomy, spleen preservation, prognosis

## Abstract

**Background:**

The feasibility of spleen-preserving distal pancreatectomy (SPDP) to treat well-differentiated non-functioning pancreatic neuroendocrine tumors (NF-pNETs) located at the body and/or tail of the pancreas remains controversial. Distal pancreatectomy with splenectomy (DPS) has been widely applied in the treatment of NF-pNETs; however, it may increase the post-operative morbidities. This study aimed to evaluate whether SPDP is inferior to DPS in post-operative outcomes and survivals when being used to treat patients with NF-pNETs in our institute.

**Methods:**

Clinicopathological features of patients with NF-pNETs who underwent curative SPDP or DPS at the First Affiliated Hospital of Sun Yat-sen University (Guangzhou, China) between January 2010 and January 2022 were collected. Short-term outcomes and 5-year survivals were compared between patients undergoing SPDP and those undergoing DPS.

**Results:**

Sixty-three patients (SPDP, 27; DPS, 36) with well-differentiated NF-pNETs were enrolled. All patients had grade 1/2 tumors. After identifying patients with T1–T2 NF-pNETs (SPDP, 27; DPS, 15), there was no disparity between the SPDP and DPS groups except for tumor size (median, 1.4 vs 2.6 cm, *P *=* *0.001). There were no differences in operation time (median, 250 vs 295 min, *P *=* *0.478), intraoperative blood loss (median, 50 vs 100 mL, *P *=* *0.145), post-operative major complications (3.7% vs 13.3%, *P *=* *0.287), clinically relevant post-operative pancreatic fistula (22.2% vs 6.7%, *P *=* *0.390), or post-operative hospital stays (median, 9 vs 9 days, *P *=* *0.750) between the SPDP and DPS groups. Kaplan–Meier curve showed no significant differences in the 5-year overall survival rate (100% vs 100%, log-rank *P *>* *0.999) or recurrence-free survival (100% vs 100%, log-rank *P *>* *0.999) between patients with T1–T2 NF-pNETs undergoing SPDP and those undergoing DPS.

**Conclusions:**

In patients with T1–T2 well-differentiated NF-pNETs, SPDP could achieve comparable post-operative outcomes and prognosis compared with DPS.

## Introduction

Provided pancreatic neuroendocrine tumors (pNETs) are rare tumors arising from neuroendocrine cells and their prevalence has been steadily rising in recent years [1]. pNETs can be classified as functioning (F-pNETs) or non-functioning (NF-pNETs) pNETs based on their ability to secrete biologically active hormones and cause characteristic symptoms [2]. Most pNETs are NF-pNETs that are often accidently discovered in patients [3]. Despite the improvement in systemic therapies, surgical resection remains the treatment of choice for patients with resectable pNETs [4]. For patients with pNETs located at the pancreatic body and/or tail, distal pancreatectomy with splenectomy (DPS) and lymphadenectomy is the recommended surgical modality. The advantages of simultaneous resection of spleen in distal pancreatectomy include facilitation of pancreatectomy and regional lymphadenectomy, hence it has been widely applied in the management of patients with pNETs at the pancreatic body and/or tail. Unfortunately, it may increase the post-operative morbidities, including thrombocytosis, elevation of thrombo-embolism risks, potential unfavorable immunological impacts, and elevation of post-operative pancreatic fistula (POPF) [5–7].

As the most common F-pNET, for insulinoma located in the pancreatic body and/or tail, enucleation or spleen-preservation distal pancreatectomy (SPDP) is feasible given its good prognosis [8, 9]. However, the feasibility of SPDP for patients with NF-pNETs located at the pancreatic body and/or tail remains controversial, since it is technique-requiring due to the difficulties in dissecting the pancreatic body and tail from the splenic vessels and hilum, and there exists fear of inadequate oncological resection.

With the development of minimally invasive techniques, especially robotic-assisted approaches, its highly magnified 3D vision field and dexterous manipulation of instruments facilitate preservation of the spleen in distal pancreatectomy [10]. However, the 5-year survival of patients with NF-pNETs at the pancreatic body and/or tail who were treated with SPDP have not been well documented.

This study aimed to compare on the outcomes between patients with NF-pNETs undergoing SPDP and those undergoing DPS in our single institute.

## Materials and methods

### Patient selection

**Figure 1.**
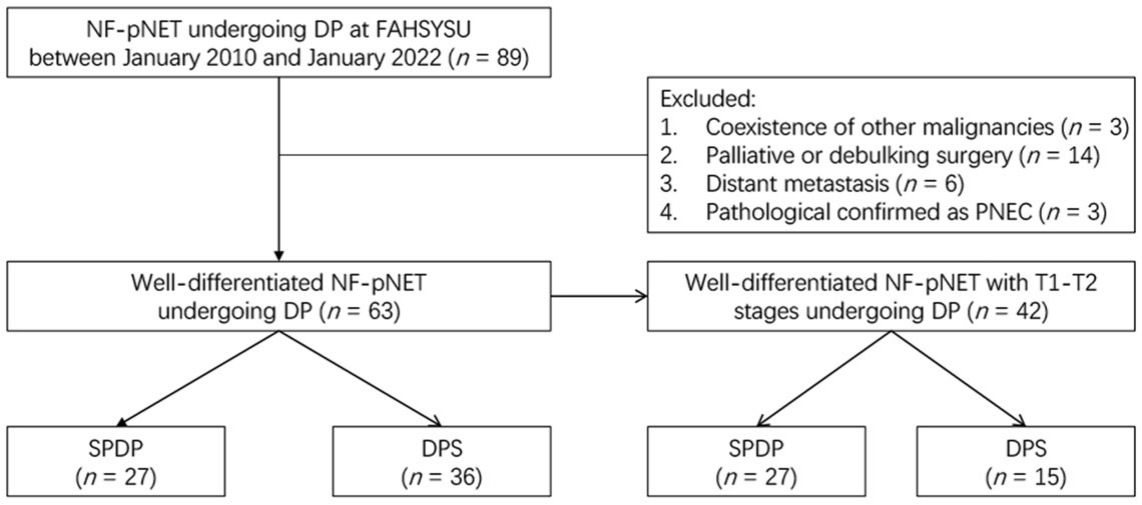
Flow chart of patient selection in this study. NF-pNET, non-functioning pancreatic neuroendocrine tumor; FAHSYSU, First Affiliated Hospital of Sun Yat-sen University; pNEC, pancreatic neuroendocrine carcinoma; SPDP, spleen-preserving distal pancreatectomy; DPS, distal pancreatectomy with splenectomy.

Patients with NF-pNETs who underwent SPDP or DPS between January 2010 and January 2022 were included in this study. The inclusion criteria were as follows: (i) preoperatively diagnosed as local pNET located at the pancreatic body and/or tail and pathologically confirmed as NF-pNET; and (ii) treated with curative distal pancreatectomy. The exclusion criteria included (i) coexistence of other malignancies; (ii) treated with palliative or debulking surgery; (iii) occurrence of synchronous liver metastasis or other distant metastasis; or (iv) pathologically confirmed as pancreatic neuroendocrine carcinoma. Finally, 63 patients with well-differentiated NF-pNETs were enrolled in this study (Figure 1). This study was approved by the Ethics Committee of the First Affiliated Hospital of Sun Yat-sen University, Guangzhou, China (Approval Number: [2022]008).

### Data collection and perioperative management

Clinicopathological data were retrospectively collected, including preoperative laboratory and imaging details, operative findings, tumor characteristics, and post-operative course. Functional status was assessed according to the presence of a detectable elevated serum level of the relevant hormone associated with a clinical syndrome. All patients were evaluated preoperatively by at least two imaging tools, including computed tomography (CT), magnetic resonance imaging (MRI), ultrasonography, or gallium 68 DOTANOC PET-CT scan.

The surgical plan was determined by a multidisciplinary team. Patients were treated with curative SPDP or DPS via open surgery or minimally invasive surgery, including laparoscopic surgery and robotic-assisted surgery. All patients were administered with prophylactic somatostatin or somatostatin analogue post-operatively. The level of drainage fluid amylase was tested on post-operative Days 1, 3, 5, and 7. Post-operative complication was evaluated according to the Clavien–Dindo classification [11]. Complications with severity of ≥grade III were defined as major complications. The definition of POPF was determined according to the 2016 International Study Group of pancreatic surgery (ISGPS) definition and the grading of post-operative pancreatic fistula [12]. Clinically relevant POPF (CR-POPF) includes grade B and grade C POPF.

All patients were followed up until death or censored at the cut-off date of May 2022. The outcomes measured were overall survival (OS) and recurrence-free survival (RFS). OS was defined as the interval between the date of surgery and the date of death or the last follow-up. Recurrence was defined as finding(s) of tumor recurrence on CT, MRI, ultrasound, or PET-CT scan.

### Statistical analysis

All statistical analyses were performed using SPSS version 24.0 software (IBM, Inc., Armonk, NY, USA). The categorical variables are presented as frequencies with percentages, whereas the continuous variables are presented as medians with interquartile range (IQR). Differences between categorical variables were compared using the chi square test or Fisher’s exact test. Differences between continuous variables were compared using the Mann–Whitney *U* test. A Kaplan–Meier curve was used to calculate the OS and RFS. Two-tailed *P *<* *0.05 was considered statistically significant.

## Results

### Comparison of clinicopathological features of patients with well-differentiated NF-pNETs undergoing SPDP and DPS

A total of 63 patients with well-differentiated NF-pNETs who underwent distal pancreatectomy were enrolled in this study, including 27 cases of SPDP and 36 cases of DPS. The clinicopathological features of patients were compared between the SPDP and DPS groups (Table 1). The SPDP group had smaller tumors than the DPS group (median of tumor size, 1.4 vs 4.4 cm, *P *<* *0.001). All patients undergoing SPDP and DPS had G1/G2 tumors; there were no G3 tumors in either group. After identifying 42 patients with T1–T2 NF-pNETs, there was no disparity between the SPDP and DPS groups except for tumor size (median, 1.4 vs 2.6 cm, *P *=* *0.001).

**Table 1.** Comparison of clinicopathological characteristics of patients with well-differentiated non-functioning pNETs undergoing SPDP and DPS

Feature	All patients			Patients with T1–T2 diseases		
	SPDP	DPS	*P*-value^a^	SPDP	DPS	*P*-value^a^
	(*n *=* *27)	(*n *=* *36)		(*n *=* *27)	(*n *=* *15)	
Age (range), years	48 (37–59)	51 (38–60)	0.906^c^	48 (37–59)	48 (33–54)	0.423^c^
Sex, no. of males (%)	15 (55.6%)	19 (52.8%)	0.827	15 (55.6%)	8 (53.3%)	0.890
BMI (range), kg/m^2^	23.1 (21.1–25.0)	23.6 (20.8–26.1)	0.662^c^	23.1 (21.1–25.0)	23.7 (18.6–26.4)	0.990^c^
ASA classification, *n* (%)			0.693^b^			0.530^b^
I+II	25 (92.6%)	32 (88.9%)		25 (92.6%)	15 (100%)	
III	2 (7.4%)	4 (11.1%)		2 (7.4%)	0 (0%)	
Diabetes, *n* (%)	3 (11.1%)	5 (13.9%)	1.000^b^	3 (11.1%)	2 (13.3%)	1.000^b^
Hypertension, *n* (%)	8 (29.6%)	6 (16.7%)	0.221	8 (29.6%)	3 (20.0%)	0.717^b^
Symptom, *n* (%)	8 (29.6%)	16 (44.4%)	0.231	8 (29.6%)	7 (46.7%)	0.270
Median of tumor size (range), cm	1.4 (1.0–2.1)	4.4 (2.7–6.0)	<0.001^c^	1.4 (1.0–2.1)	2.6 (1.8–3.0)	0.001^c^
AJCC T stage, *n* (%)			<0.00^b^			NA
T1–T2	27 (100%)	15 (41.7%)		27 (100%)	15 (100%)	
T3–T4	0 (0%)	21 (58.3%)		0 (0%)	0 (0%)	
WHO grade, G1/G2, *n* (%)	27 (100%)	36 (100%)	NA	27 (100%)	15 (100%)	NA
LVI, *n* (%)	3 (11.1%)	8 (22.2%)	0.326^b^	3 (11.1%)	2 (13.3%)	1.000^b^
Neural invasion, *n* (%)	0 (0%)	3 (8.3%)	0.253^b^	0 (0%)	0 (0%)	NA
Vascular invasion, *n* (%)	0 (0%)	4 (11.1%)	0.128^b^	0 (0%)	0 (0%)	NA

a Chi square test.

b Fisher’s exact test.

c Mann–Whitney *U* test.

pNET, pancreatic neuroendocrine tumor; SPDP, spleen-preserving distal pancreatectomy; DPS, distal pancreatectomy with splenectomy; BMI, body mass index; ASA, American Society of Anesthesiologists; AJCC, American Joint Committee on Cancer; WHO, World Health Organization; LVI, lymph-vascular invasion; NA, not available.

### Comparison of short-term outcomes of patients with well-differentiated NF-pNETs undergoing SPDP and DPS

The operative details and short-term outcomes of patients with NF-pNETs were compared between the SPDP and DPS groups (Table 2). In patients with T1–T2 NF-pNETs, the minimally invasive rate was 100% in the SPDP group (laparoscopic surgery, 29.6%; robotic-assisted surgery, 70.4%) and 80.0% in the DPS group (laparoscopic surgery, 20.0%; robotic-assisted surgery, 60.0%), with a conversion rate of 3.7% (1/27) and 16.7% (2/12) in the SPDP and DPS groups, respectively. All the spleen-preserving procedures were performed via the Kimura technique. There were no differences in operation time (median, 250 vs 295 min, *P *=* *0.478), intraoperative blood loss (median, 50 vs 100 mL, *P *=* *0.145), blood transfusion (3.7% vs 6.7%, *P *=* *1.000), post-operative complications with severity of ≥Clavien–Dindo Grade III (3.7% vs 13.3%, *P *=* *0.287), CR-POPF (22.2% vs 6.7%, *P *=* *0.390), or post-operative hospital stays (median, 9 vs 9 days, *P *=* *0.750) between the SPDP and DPS groups. There was no mortality in either group. Fewer lymph nodes were obtained in the SPDP group than in the DPS group (median, 0 vs 2, *P *=* *0.013), but there was no significant difference in the rate of lymph-node metastasis (LNM) between the two groups (3.7% vs 6.7%, *P *=* *1.000).

**Table 2.** Comparison of operative details and short-term outcomes of patients with well-differentiated non-functioning pNETs undergoing SPDP and DPS

Feature	All patients			Patients with T1–T2 disease		
	SPDP	DPS	*P*-value^a^	SPDP	DPS	*P*-value^a^
	(*n *=* *27)	(*n *=* *36)		(*n *=* *27)	(*n *=* *15)	
Surgery approach, *n* (%)			0.002^b^			0.076^b^
Open surgery	0 (0%)	12 (33.3%)		0 (0%)	3 (20.0%)	
Laparoscopic surgery	8 (29.6%)	6 (16.7%)		8 (29.6%)	3 (20.0%)	
Robotic-assisted surgery	19 (70.4%)	18 (50.0%)		19 (70.4%)	9 (60.0%)	
Operation time, min	250 (195–340)	290 (206–356)	0.266^c^	250 (195–340)	295 (205–375)	0.478^c^
Intraoperative blood loss (range), mL	50 (50–100)	125 (50–200)	0.015^c^	50 (50–100)	100 (50–200)	0.145^c^
Blood transfusion, *n* (%)	1 (3.7%)	3 (8.3%)	0.629^b^	1 (3.7%)	1 (6.7%)	1.000^b^
Major complications^d^, *n* (%)	1 (3.7%)	5 (13.9%)	0.226^b^	1 (3.7%)	2 (13.3%)	0.287^b^
CR-POPF, *n* (%)	6 (22.2%)	4 (11.1%)	0.303^b^	6 (22.2%)	1 (6.7%)	0.390^b^
Mortality, *n* (%)	0 (0%)	0 (0%)	NA	0 (0%)	0 (0%)	NA
Post-operative stay (range), days	9 (8–11)	10 (9–12)	0.416^c^	9 (8–11)	9 (8–11)	0.750^c^
Number of lymph nodes examined (range)	0 (0–1)	2 (0–7)	0.009^c^	0 (0–1)	2 (1–7)	0.013^c^
Lymph-node metastasis, *n* (%)	1 (3.7%)	2 (5.6%)	1.000^b^	1 (3.7%)	1 (6.7%)	1.000^b^

a Chi square test.

b Fisher’s exact test.

c Mann–Whitney *U* test.

d Post-operative complications with severity of ≥Clavien–Dindo grade III.

pNET, pancreatic neuroendocrine tumor; SPDP, spleen-preserving distal pancreatectomy; DPS, distal pancreatectomy with splenectomy; CR-POPF, clinically relevant post-operative pancreatic fistula; NA, not available.

### Comparison of survival of patients with well-differentiated pNETs undergoing SPDP and DPS

**Figure 2.**
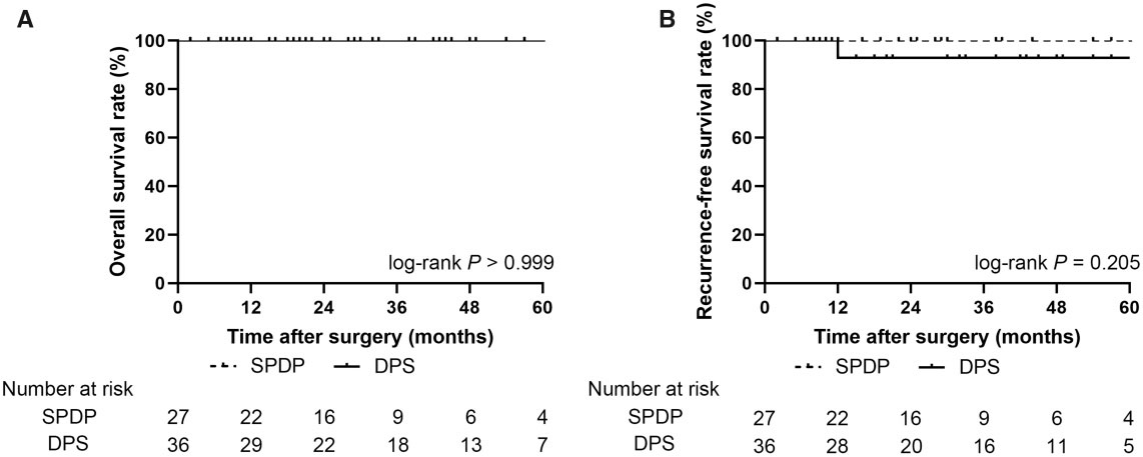
Kaplan–Meier curve of overall survival (A) and recurrence-free survival (B) in patients with well-differentiated NF-pNETs treated with SPDP and DPS. NF-pNET, non-functioning pancreatic neuroendocrine tumor; SPDP, spleen-preserving distal pancreatectomy; DPS, distal pancreatectomy with splenectomy.

**Figure 3.**
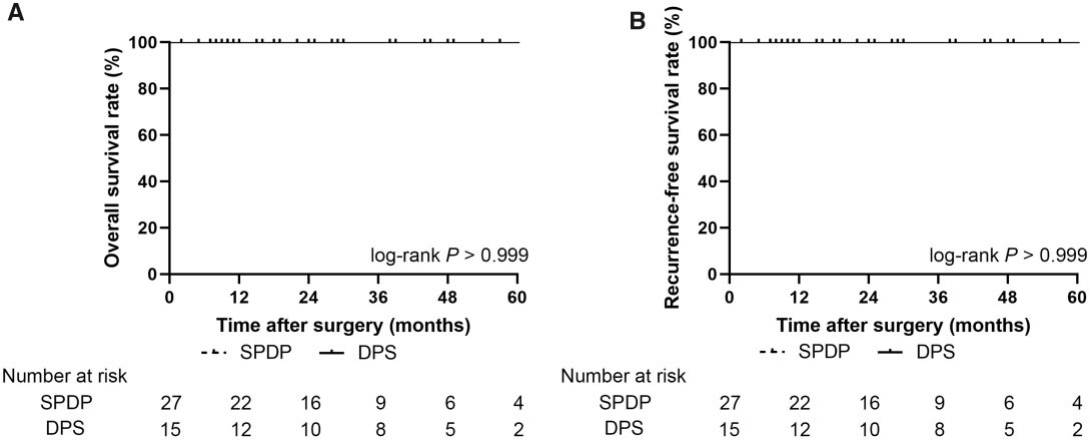
Kaplan–Meier curve of overall survival (A) and recurrence-free survival (B) in patients with well-differentiated T1–T2 NF-pNETs treated with SPDP and DPS. NF-pNET, non-functioning pancreatic neuroendocrine tumor; SPDP, spleen-preserving distal pancreatectomy; DPS, distal pancreatectomy with splenectomy.

The median follow-up time was 28.1 months. The median OS and RFS were not reached in either group. A Kaplan–Meier curve showed no significant differences in the 5-year OS (100% vs 100%, log-rank *P *>* *0.999) or 5-year RFS (100% vs 92.9%, log-rank *P *=* *0.205) between patients with NF-pNETs undergoing SPDP and those undergoing DPS (Figure 2). Subgroup analysis showed no significant difference in 5-year OS (100% vs 100%, log-rank *P *>* *0.999) or 5-year RFS (100% vs 100%, log-rank *P *>* *0.999) between patients with T1–T2 NF-pNETs undergoing SPDP and those undergoing DPS (Figure 3).

## Discussion

Distal pancreatectomy can be performed combined with splenectomy or spleen preservation. Several studies have shown the feasibility and potential benefits of preserving the spleen in distal pancreatectomy, including protecting the immune function and reducing the risk of overwhelming post-splenectomy infection, intraoperative blood loss, post-operative infections, and other complications due to splenectomy [13–15]. However, in order to avoid inadequate tumor resection, a spleen-preservation procedure was not applied in high-malignant lesions such as pancreatic adenocarcinoma [16]. In contrast, the use of SPDP for pre-cancerous or low-grade tumors such as pNETs remains controversial. A systemic review showed that 9%–16% of SPDPs were performed to treat patients with pNETs [17]. However, as the most common type of pNET, few studies focus on the value of SPDP in the long-term outcomes of NF-pNET patients such as OS and recurrence. Therefore, this study aimed at comparing the therapeutic value of SPDP with DPS in well-differentiated NF-pNETs in our single institute. After identifying patients with T1–T2 NF-pNETs, there was no disparity between the SPDP and DPS groups except for tumor size. There were no significant differences in the intraoperative or post-operative outcomes between the SPDP and DPS groups, which was comparable to the result of a previous report [18]. Survival analysis showed no significant differences in 5-year OS and RFS between patients with T1–T2 NF-pNETs undergoing SPDP and those undergoing DPS. Since the median tumor diameter differed by only 1.2 cm between the two groups, this study still demonstrated the safety and feasibility of SPDP in patients with T1–T2 NF-pNETs.

Regional lymphadenectomy is commonly recommended during resection of pNETs due to the potential risk of LNM. Although some studies demonstrated the prognostic value of the total number of lymph nodes examined (TNLE) and LNM in pNETs [19, 20], other research indicated that TNLE and LNM may have less important prognostic value in pNETs with some favorable characteristics (such as tumor ≤2 cm or Ki-67 index <3%) [21]. In this study, the median TNLE in patients undergoing SPDP was 0, which was significantly lower than in patients undergoing DPS in this study or those in previous studies [18]. Although achieving more lymph nodes can help with more accurate staging and avoid false-negative findings [22, 23], previous research proposed that the prevalence of LNM in pNETs is related to tumor size and tumor grade (Ki-67 index) [19, 24, 25], which indicated that LNM was unlikely to occur in patients with small G1 pNETs. In this study, since most of the patients treated with SPDP had G1 pNETs of <2 cm (19/27, 70.4%), the possibility of actual LNM might be low, which was also confirmed by the fact that only one patient had LNM. It suggested that SPDP may reduce the TNLE in patients with pNETs, resulting in insufficient accuracy of nodal staging. Nevertheless, no recurrence or tumor-related death occurred in all patients with T1–T2 NF-pNETs in this study. The significance of lymphadenectomy and TNLE in early-stage NF-pNETs needs to be further investigated.

In this study, the 5-year OS and 5-year RFS of patients with T1–T2 NF-pNETs undergoing SPDP and DPS were 100%, which suggested that SPDP can achieve an ideal oncologic prognosis in patients with early-stage NF-pNETs when compared with the results of previous literature [18]. No recurrence or metastasis was observed in patients with T1–T2 NF-pNETs in this study. It is reasonable that recurrence may occur in patients with higher Ki-67 or larger tumors; therefore, higher Ki-67 or larger tumors were also considered poor prognostic factors in the recurrence and metastasis of pNETs after surgery. However, in the current study, even in some patients with a tumor size of ≥2 cm (7/27, 25.9%), a good prognosis can still be obtained after spleen preservation. Therefore, SPDP can achieve a comparable RFS and OS to DPS for NF-pNET patients if technically feasible.

This study revealed that SPDP can ensure radical resection of the tumor while preserving the spleen function in patients with T1–T2 well-differentiated NF-pNETs. Although the perioperative outcomes and 5-year survival of SPDP in pNET patients are acceptable, there are still some pNET patients who may be unsuitable for SPDP. Because all spleen-preservation procedures were performed via the Kimura technique in our institute, SPDP should not be considered for patients whose tumors invade the splenic vessels or with suspected splenic hilar LNM.

This study has several limitations. First, this study is a single-center, retrospective study with a relatively small sample size, which may lead to biased results. Second, although all SPDPs were performed using the Kimura technique in our center, SPDP may not be recommended for patients with large tumors at the tail of the pancreas. Differences in two spleen-preservation methods (Kimura and Warshaw techniques) for pNETs can be compared in the future. In addition, since most of the patients treated with SPDP had small G1/G2 pNETs, while most of the pNET patients undergoing DPS had T3–T4 tumors, it is difficult to match patients treated with SPDP and DPS to evaluate the difference between the two procedures.

In conclusion, this study revealed that although the number of examined lymph nodes may be lower in patients receiving SPDP than in those receiving DPS, the short-term outcomes and 5-year survival of patients with well-differentiated non-functioning pNETs who underwent SPDP was acceptable, especially in those with T1–T2 diseases. In addition, SPDP could preserve the function of the spleen and might improve the patients’ quality of life post-operatively. For small NF-pNETs with low risk of LNM, SPDP could be considered.

## Authors’ Contributions

Conception and design: X.Y.Y. Collection and assembly of data: X.T.H., J.Z.X., J.P.C., P.F., C.S.H., W.C., L.J.L. Data analysis and interpretation: X.T.H., J.Z.X., X.Y.Y. Manuscript writing and revision: X.T.H., J.Z.X., X.Y.Y. All authors read and approved the final manuscript.

## Funding

None.
